# Characterizing health care utilization following hospitalization for a traumatic brain injury: a retrospective cohort study

**DOI:** 10.1080/02699052.2020.1861650

**Published:** 2020-12-24

**Authors:** Johanne Eliacin, Ziyi Yang, Jacob Kean, Brian E. Dixon

**Affiliations:** a Center for Health Information and Communication, Richard L. Roudebush VA Medical Center, Indianapolis, USA; b Department of Psychology, Indiana University-Purdue University - Indianapolis, Indianapolis, USA; c Health Services Research, Regenstrief Institute, Inc., Indianapolis, USA; d Department of Biostatistics, Indiana University-Purdue University - Indianapolis, Indianapolis, USA; e Informatics, Decision-Enhancement and Analytic Sciences Center, Health Services Research and Development, VA Salt Lake City Health Care System, Salt Lake City, USA; f Department of Population Health Sciences, University of Utah School of Medicine, Salt Lake City, USA; g Department of Communication Sciences and Disorders, University of Utah School of Medicine, Salt Lake City, USA; h Department of Epidemiology, Indiana University Richard M. Fairbanks School of Public Health, Indianapolis, USA; i Center for Biomedical Informatics, Regenstrief Institute, Indianapolis, USA

**Keywords:** Health services utilization, traumatic brain injury, superutilizers, state-wide registry

## Abstract

**Objective:**

The purpose of this study was to characterize health services utilization among individuals hospitalized with a traumatic brain injury (TBI) 1-year post-injury.

**Methods:**

Using a retrospective cohort design, adult patients (n = 32, 042) hospitalized with a traumatic brain injury between 2005 and 2014 were selected from a statewide traumatic brain injury registry. Data on health services utilization for 1-year post-injury were extracted from electronic medical and administrative records. Descriptive statistics and logistic regression were used to characterize the cohort and a subgroup of superutilizers of health services.

**Results:**

One year after traumatic brain injury, 56% of participants used emergency department services, 80% received inpatient services, and 93% utilized outpatient health services. Superutilizers had ≥3 emergency department visits, ≥3 inpatient admissions, or ≥26 outpatient visits 1-year post-injury. Twenty-six percent of participants were superutilizers of emergency department services, 30% of inpatient services, and 26% of outpatient services. Superutilizers contributed to 81% of emergency department visits, 70% of inpatient visits, and 60% of outpatient visits. Factors associated with being a superutilizer included sex, race, residence, and insurance type.

**Conclusions:**

Several patient characteristics and demographic factors influenced patients’ healthcare utilization post-TBI. Findings provide opportunities for developing targeted interventions to improve patients’ health and traumatic brain injury-related healthcare delivery.

## Introduction

Traumatic brain injury (TBI) is a form of acquired brain injury that occurs when a sudden trauma or impact to the head causes damage to the brain. Advances in treatment of TBI have improved the mortality rate for TBI (1,2). Yet, TBI remains a leading cause of mortality and disability (1). TBI contributes up to a third (30.5%) of all injury-related deaths and accounts for 50,000 deaths every year in the U.S (1). While many people with TBI recover from their injuries, a subset of TBI survivors experience persistent and sometimes lifelong disabilities (1,3–6).

Due to the lasting effects of TBI on the brain, researchers and clinicians are increasingly treating TBI as a chronic health condition that may require long-term care (3,5). It is estimated that up to 5.3 million persons in the United States are living with a TBI-related disability (1,6). Long-term disability associated with TBI not only reduces quality of life for the patient, but also results in prolonged medical, economic, and social costs (4,7–10). Yet, the healthcare trajectories and health services utilization of persons living with TBI sequelae are poorly understood (4,11).

Barriers to understanding the impacts and outcomes of TBI include lack of integrated health records and studies with small sample sizes that often rely on self-report (12,13). Moreover, studies focusing on healthcare utilization related to TBI are limited, which undermines the generation of reliable results to inform interpretations of healthcare utilization over time and across the continuum of TBI severity (14–16). To address these challenges and facilitate cross-study comparisons, we sought to characterize patterns of health services utilization of patients who were hospitalized with a TBI 1-year post-injury using a statewide registry with integrated electronic medical records and claims data from multiple health systems.

## Materials and methods

### Data source

Data originated from the Indiana Network for Patient Care (INPC). INPC is one of the largest community-based networks of electronic health records in the United States (17,18). It connects over 100 health care facilities, including hospitals, physicians’ practices, pharmacy networks, long-term post-acute care facilities, laboratories, and radiology centers, representing 38 distinct health systems across the state. INPC maintains over 13 billion structured observations for over 19 million individuals, living and deceased, and includes claims records from public as well as private payers. INPC is representative of the state’s population with respect to age, gender, and race. The INPC captures data on more than two-thirds of individuals living in Indiana (19) and reflects the population that receives health care services in the state.

Data on all individuals (adults and children) diagnosed with mild, moderate, and severe TBI were extracted from INPC and entered into a statewide TBI registry, the Indiana TBI Registry (20,21). The statewide registry contains demographic data (e.g., age), co-morbidity data (e.g., ICD diagnoses), healthcare utilization data (e.g., visit type, inpatient admission and discharge disposition), laboratory testing data, and medication history (e.g., drug name) extracted from electronic medical records and administrative data. Moreover, patients’ health records in the registry are linked together to provide comprehensive health information on patients with a diagnosis of TBI and to enable longitudinal analysis by patient or sub-population (21).

### Selection of study population: inclusion and exclusion criteria

For this study, we extracted data from the Indiana TBI Registry from 01/01/2005 to 12/31/2014 on adults hospitalized with a TBI. We examined healthcare utilization data for these patients for a period of 1 year (≤ 365 days) following their index TBI event, described as the first occurrence of a TBI-related ICD code in an individual’s electronic health record. TBI diagnosis was determined using the International Classification of Diseases, Ninth Revision, Clinical Modification (ICD-9-CM) at the time of visit (800.00–801.99, 803.00–804.99, 850.00–850.99, 950.1–950.3, 959.01, 995.55). Given that traditional measures of severity, such as Glasgow Coma Scale scores, were missing in the INPC data, we limited our sample to patients who were hospitalized within 48 hours of the index TBI event. Because they were hospitalized, these patients likely represent individuals with a moderate-to-severe TBI.

Patients were eligible to be included in the study if they a) were hospitalized within 48 hours of sustaining an index TBI event, b) did not die on the same date of their index admission with a TBI, and c) were 18 years or older at the time of their TBI.

The following variables were extracted from each patient’s health records: (1) demographic information (e.g., age, insurance type, and urban vs. rural residency status) and (2) health care utilization data (e.g., diagnostic codes, dates of admission and discharge). This study was approved by the Indiana University Institutional Review Board.

### Statistical methods

All analyses were conducted in SAS version 9.4. Patient characteristics were summarized using descriptive statistics. For continuous variables, we calculated mean and standard deviation as well as median and IQR if the distribution was skewed. For categorical variables, we calculated frequency and percentage. We calculated Charlson Comorbidity Index Score using patients’ history of comorbid conditions one year prior to their TBI. Due to the skewed distribution of Charlson Comorbidity Index Score, we dichotomized the scores into two categories of presence or absence of prior comorbid conditions. We used False Discovery Rate and Hochberg methods to adjust for multiple comparisons (22).

Our primary outcomes of interest were the number of inpatient admissions, emergency department (ED) visits, and outpatient encounters for all participants one- year post-TBI. In subsequent analyses, we examined health services utilization for 1-year post-TBI to identify superutilizers – patients with high utilization rates of health services. We used the 75% quartile of the total number of visits as the cutoff point for super utilization of health services. We considered the 90% decile of total visits as the cutoff point for extreme utilization of health services. Patients whose number of visits were equal to or greater than either threshold were identified as super-utilizers and/or extreme users, respectively. These terms and their calculations are consistent with prior health services research (23–25). We generated frequencies for superutilizers and extreme users by each type of visit (also referred to as care setting). We further calculated descriptive statistics for each group.

The demographic characteristics were compared between superutilizers and non-superutilizers. To examine the factors associated with being superutilizers, we conducted multivariate logistic regression using the following model: *logit*(*p*) age + sex + residency + race + insurance + health services utilization (the number of inpatient admissions, ED and outpatient visits) + presence of any comorbidity one year prior to TBI. The covariates were selected based on clinical knowledge of the patient population and the outlying values were analyzed based on the summary statistics. Patients with missing information for each of the covariates were categorized as “Unknown.”. The AUC under the ROC were reported for the accuracy of model (26). We repeated these procedures for the extreme utilizers.

## Results

### Participants

**Figure 1.**
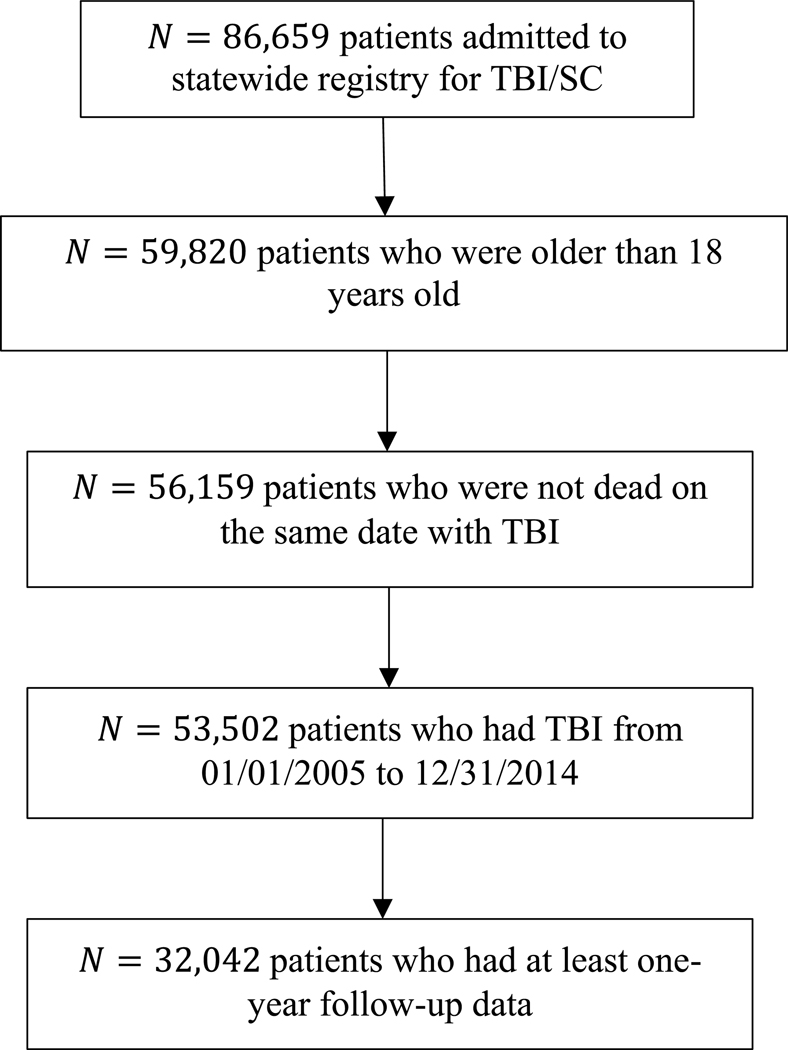
Consort diagram of participants’ selection in the study cohort from the statewide TBI registry.

**Figure 2.**
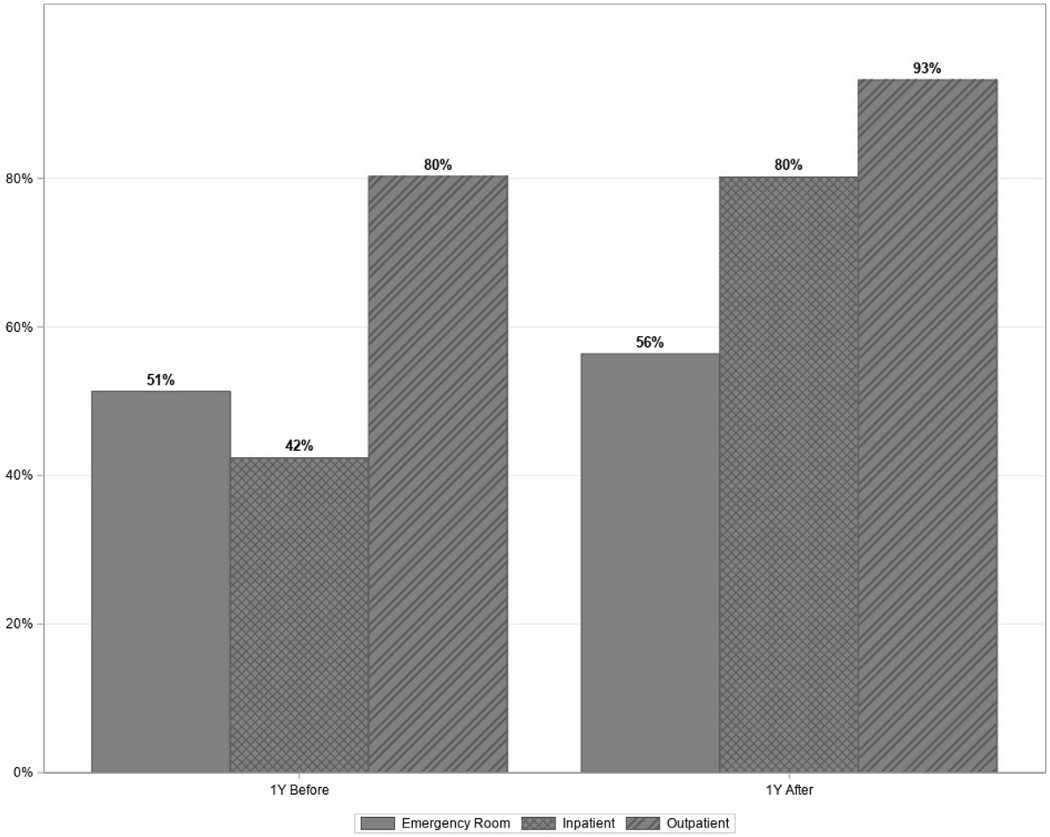
Distribution of participants’ Emergency Department (ED) visits, inpatient admissions, and outpatient healthcare visits 1 year before and 1 year after index TBI (N = 32,042).

Figure 1 summarizes data for the cohort and its selection from the statewide TBI registry. The cohort consisted of 32,042 patients, representing 40% of patients in the full registry. The proportion of patients who had inpatient admission increased from 42% (n = 13,582) prior to TBI to 80% (n = 25,695) within a year thereafter. Similarly, the proportions of patients who had ED and outpatient admission increased from 51% (n = 16,442) to 56% (n = 18,060), and 80% (n = 25,725) to 93% (n = 29,886), respectively (See Figure 2).

### Baseline characteristics of study participants and superutilizers of all three visit types

As shown in Table 1, participants’ average age at the time of the TBI was 53.5 years (SD = 21.4). Most patients were female (55%), White (63%), and urban dwellers (73%). Based on the most recent insurance information available at the index admission date in the patient’s medical records, most patients were uninsured or had unknown insurance status (63%). The average length of stay of the index TBI inpatient admission was 4 days. Approximately 40% of patients had a history of comorbid conditions one year prior to their TBI (Charlson Comorbidity Index Score of 1 or greater). Baseline characteristics of superutilizers of all three visit types were similar. Superutilizers had an average age of 51.3 (SD 19.6), were mostly female (61%), White (72%), and urban dwellers (73%). Their average length of stay of the index TBI admission was 4 days. They were also more likely to have comorbid conditions prior to the TBI index event (64%).

### Distribution of superutilizers and extreme users

Based on the 75% quartile cutoff point, superutilizers possessed 3 or more ED visits, at least 3 inpatient admissions or a minimum of 26 outpatient visits 1 year post-injury. Twenty-six percent of participants were superutilizers of ED visits, 30% were superutilizers of inpatient admissions, and 26% were superutilizers of outpatient services. Superutilizers contributed to 81% of ED visits, 70% of inpatient visits, and 60% of total outpatient healthcare services. Nearly 8% of the patients (n = 2560) were superutilizers of all three visit types.

We also identified extreme users of healthcare services post-TBI using a 90% decile cutoff point. Based on this cutoff point, participants with 6 or more ED visits (11% of cohort), at least 5 inpatient admissions (13% of cohort), or a minimum of 41 outpatient visits (10% of cohort) were identified as extreme users of health services. These extreme users accounted for 56% of ED visits, 45% of inpatient admissions, and 32% of outpatient visits.

### Characteristics of superutilizers of health services post-TBI based on 75% quartile

Table 2 summarizes the characteristics of superutilizers by each health care setting. Briefly, compared to non-superutilizers, superutilizers across all visit types were female, had a history of comorbid conditions 1 year prior to their TBI, and possessed higher mortality (*p* <.001). Specific co-morbid condition trends were consistent across the top 5 conditions. Superutilizers of ED visits and inpatient admissions were younger at the time of their TBI compared to non-superutilizers (49.5 vs. 54.9 years and 52.8 vs. 53.8 years respectively, *p* < .001), whereas superutilizers of outpatient visits were older (56.1 vs. 52.6 years old, *p* < .001). Superutilizers of inpatient admissions had longer index inpatient stays than non-superutilizers (4.5 vs. 3.5 respectively, *p* < .001).

### Characteristics of extreme users of health services post-TBI based on 90% decile

Characteristics of extreme users based on the 90% cutoff point are summarized in Supplementary Table S1. They are similar to those of superutilizers of health services. However, the age difference between extreme users and non-extreme users of ED visits and inpatient admissions is more pronounced compared to those of superutilizers and non-superutilizers described above (45.7 vs. 54.5 and 49.5 vs. 54.1 years respectively, *p* < .001). Also, extreme users of ED visits were about 4 years younger than superutilizers of ED visits at the time of their index TBI event (45.7 vs 49.5). Similarly, extreme users of inpatient admissions had an average age of 49.5 at the index TBI event compared to superutilizers who had an average age of 52.8. In contrast, extreme users of outpatient health services were older than those who were not (56.2 vs. 53.2 years old, *p* < .001).

### Factors associated with being superutilizers and extreme users of health services post-TBI

Table 3 summarizes the factors associated with superutilization based on multivariate logistic regression. The area under the curve was.80 for factors associated with being a superutilizer of ED visits indicating good accuracy of the logistical model, and .69 for being a superutilizer of inpatient admission and .77 for outpatient admission, suggesting a fair accuracy of the model. Supplementary Table S2 shows factors associated with extreme utilization based on 90% decile.

#### A. Factors associated with being a superutilizer of ED visits

Several factors were associated with superutilization of ED visits post-TBI. Patients aged 18–24 years (OR = 1.39, 95% CI 1.25–1.54), 25–44 years (OR = 1.58, 95% CI 1.46–1.72), and 45–64 years (OR = 1.29, 95% CI 1.20, 1.39) were significantly more likely to be a superutilizer of ED visits post-TBI compared with those over 65 years (*p* < .001). Prior ED utilization and comorbidity were also strongly associated with ED super-utilization. Patients with ED visits in the year prior to their index TBI event were at higher odds of being an ED super-utilizer post-TBI (OR = 1.44, 95% CI 1.42–1.46, *p* < .001). Moreover, patients with a Charlson comorbidity index >1 in the year prior to their index TBI event were at higher odds of being a superutilizer of ED visits post-TBI (OR = 1.35, 95% CI 1.27–1.45, *p* < .001).

#### B. Factors associated with being a superutilizer of inpatient admissions

Several factors were associated with superutilization of inpatient visits post-TBI. Like the ED setting, prior inpatient utilization and comorbidity were strongly associated with inpatient superutilization. Patients with inpatient visits in the year prior to their index TBI event were at higher odds of being an inpatient superutilizer post-TBI (OR = 1.31, 95% CI 1.29–1.33, *p* < .001). Furthermore, patients with a Charlson comorbidity index >1 in the year prior to their index TBI event were at higher odds of being a superutilizer of inpatient visits post-TBI (OR = 1.44, 95% CI 1.36–1.53, *p* < .001). Participants aged 18 to 24 were less likely to be superutilizers than those aged 25 to 44 (OR = 0.83, 95% CI 0.76–0.91, *p* < .001) and 45 to 64 (OR = 0.83, 95% CI 0.76, 0.92, *p* < .001). Nonwhite patients were at higher odds of being a superutilizer of inpatient services comparted to White patients (OR = 1.13, 95% CI 1.04, 1.21, *p* = .002). Patients with private or commercial health insurance were at lower odds of being superutilizers than those with public health insurance (OR = 0.57, 95% CI 0.52–0.63, *p* < .001).

#### C. Factors associated with being a superutilizer of outpatient visits

Patients aged 18 to 24 were at lower odds of being a superutilizers of outpatient visits compared to those who were in the 25–44 age group (OR = 0.67, 95% CI 0.60–0.75) and those aged 45 to 64 (OR = 0.67, 95% CI 0.60–0.75, *p* < .001). The odds of being a superutilizer of outpatient visits were lower by 10% for female patients compared to male patients (OR = 0.90, 95% CI 0.84–0.95, *p* < .001).

## Discussion

This study characterizes health services utilization 1-year post-injury among patients hospitalized with TBI using a large scale, statewide database that includes patient records from multiple health systems. Our study offers several insights. First, although demographic characteristics of patients with TBI were largely similar to those of patients in previous studies (1,27,28), most of the patients in this study were women. This is contrary to prior work indicating a higher incidence of TBI among men (14,15,27,29). Indeed, as past studies indicated, men may be at higher risk of TBI compared to women because of injuries sustained in high risk activities, behaviors, and occupations, such as professional contact sports, construction, or military occupations, and used of firearms in injuries (30–32). Whereas this study focuses on civilians and reflects the general state population. Furthermore, women in this study were less likely to be superutilizers of outpatient healthcare but were more likely to be superutilizers of ED services. Reports on sex differences post-TBI have been inconsistent (33–38). For example, some studies have examined the role of estrogen as a neuroprotective factor, moderating the impact of brain injury in women, while others have explored the functional outcomes of TBI among women in terms of their reduced participation in the workforce post-TBI (39,40). The intersection of sex and gender may affect female patients’ health, social conditions, role expectations, and use of health services post-injury. Given limited research on sex differences among patients with TBI (41,42), future investigations into clinical and social factors that contribute to women’s post-TBI health and health services utilization are needed.

Second, a substantial percentage of patients in this cohort were characterized as superutilizers and extreme users. In a prior TBI study (43), superutilizers of TBI-related services were defined as individuals who had 3 or more encounters per year 1-year post-TBI. These reports are consistent with our findings for superutilizers of health services 1-year post injury. However, unlike our study, Salisbury and colleagues (43) did not differentiate the number of encounters per visit type. In our study cohort, more than 26% of patients were characterized as superutilizers of ED, inpatient, or outpatient services. Also, more than 10% of patients were identified as extreme users of health services post-TBI. In contrast, super-utilizers in the general population represent 3% to 8% of individuals that accumulate multiple ED visits and hospital admissions (44–46). This figure is similar, however, to the 8% of the cohort that were superutilizers across all three visit types (ED, inpatient admissions, and outpatient visits).

Additionally, an evolving area of health services research focuses on identifying who are high-cost and high-level healthcare service users (47) and how to efficiently meet the needs of these group while reducing healthcare expenditures (48–50). As such, super-utilizers or high service users are often discussed in the context of understanding the clinical groups of these patients, the contexts of their interactions with health services, and the financial implications of their utilizations on the healthcare system (51–53).

Policy initiatives and interventions developed to reduce the number of superutilizers often seek to decrease costly use of health services through prevention of inpatient admissions and unnecessary use of ED services (54–56). In the context of TBI, there are limited data on patterns of healthcare utilization for TBI survivors and no specific guidelines on what types and number of visits are expected or should occur after hospitalization for brain injuries. Therefore, our characterization of superutilizers provides a foundation for understanding healthcare utilization of these populations upon which we can develop future guidelines for treatment and health services.

Third, several demographic factors were associated with being a superutilizer of health services such as, patients’ age, sex, and health insurance status. Younger patients were consistently more likely to be superutilizers of the ED and inpatient visits compared to older groups. Extreme users of ED and inpatient visits were also younger compared to their counterparts. In fact, the age difference between extreme users and non-extreme users was more pronounced than the age difference between superutilizers and non-superutilizers, with extreme users being significantly younger. Yet this trend did not hold for outpatient utilization. This is surprising, as prior research found that older patients with more comorbidities require more intensive care (16) and are more likely to have high utilization of health services before and after TBI (16,57,58). Yet this finding is consistent with a study by McFarlane et al. (59) which found that younger adults (aged 18 to 44 years) with serious TBI were at higher risk for acute ischemic stroke within 180 days after TBI. Although the reasons are unknown, it is possible that younger patients have fewer available resources (e.g., health literacy, established primary care provider) and rely more on emergency services for healthcare. Given the high cost associated with ED and inpatient services, these findings warrant future investigations to help identify better ways to support young adults with TBI and to reduce preventable healthcare utilization.

Lastly, race (White/nonwhite) may influence health service utilization. Nonwhite patients had lower odds of being super-utilizers of outpatient services compared to White patients. The opposite was true for inpatient services where nonwhite patients were at higher odds of being superutilizers. These findings are similar to prior studies, which are also inconsistent. Although several studies have reported racial disparities in the risks, health outcomes, and mortality associated with TBI, few have examined racial differences in health services utilization post-TBI (60). Existing studies that have examined race and health services utilization have reported that minority patients are less likely to use hospital and outpatient health services post-TBI (4,61). Whereas other studies show no significant relationship between race and health care utilization among patients with TBI (62). Our findings lend further support for establishing a benchmark for healthcare services utilization and facilitating equitable access to services for minority patients with TBI.

### Study limitations

There are several limitations to note. The Indiana TBI Registry may not capture all cases of TBI in the state, which limits the generalizability of our findings to the referent population. Several factors may have affected the content of the TBI registry, our cohort composition, and data analyses, including missing data, institutions reporting structures, as well as leakage or loss of records to follow-up. For example, our database does not include data from nonparticipating facilities and some small, private outpatient practices. Moreover, participating institutions do not have a uniform reporting schedule and structure. Therefore, missing data from these institutions may also affect findings. Further, we likely do not have data from patients who relocated to other states or who utilized services at institutions that do not report to INPC. The loss of patients’ records to follow-up introduces biases in the analyses because of potential differences between patients who were included in the statewide registry and those who were not. Also, because this is a retrospective study, it is difficult to ascertain the reason for the loss of patients to follow-up.

Another limitation of this study is that health insurance data were limited to information gathered near the time of hospitalization for the index TBI event. It is possible that some patients may experience a change in health insurance status post TBI, especially if they experience a change in employment status. Lastly, due to limitations of our dataset, we were not able to differentiate whether it was the TBI due to its cause or severity, or other contextual factors associated with the index event that drove patients’ healthcare utilization. While many patients hospitalized with a diagnosis with TBI may have sustained a moderate to severe brain injury, it is possible that other factors such as other injuries and medical conditions may have influenced patients’ need for subsequent healthcare services including inpatient admissions. Therefore, more research is needed to better understand drivers of healthcare utilization in this patient population.

## Conclusion

Despite its limitations, this study has several strengths. The study’s statewide data, though limited, provide adequate statistical power to detect differences, allowing for better understanding of patient characteristics and subgroups of patients that may contribute to healthcare utilization patterns. It further identifies and characterizes superutilizers for health services post-TBI, which may provide the first step for understanding and assessing the healthcare needs of patients.

## Data availability

The data that support the findings of this study are available from the Regenstrief Institute which serves as the honest data broker for the Indiana Health Information Exchange. Restrictions apply to the availability of these data, which were used under a data use agreement for this study. Data are available from the authors [BD] with the permission of the Regenstrief Institute.

## Figures and Tables

**Table 1.** Baseline characteristics of TBI patients admitted to hospital from 2005–2014 across multiple hospitals in Indiana, United States (N = 32,042), and of superutilizers of all three visit types (ED, inpatient admissions, and outpatient visits), (N = 2560).

Baseline characteristics of cohort participants (N = 32,042)		Baseline characteristics of Superutilizers of all three visit types (N = 2560)	

Patient Characteristic	Total (N = 32,042)	Patient Characteristic	Total

Age at TBI, Mean (SD)	53.5 (21.4)	Age at TBI, Mean (SD)	51.3 (19.6)
Length of Stay of the INDEX Inpatient Visit (Days), Mean (SD)	3.8 (15.6)	Length of Stay of the INDEX Inpatient Visit (Days), Mean (SD)	4.0 (8.5)
Length of Stay of the INDEX Inpatient Visit, Median (IQR)	1 (1, 4)	Length of Stay of the INDEX Inpatient Visit, Median (IQR)	1.0 (1.0, 4.0)
Age at TBI, N (%)		Age at TBI, N (%)	
18 ~ 24	3607 (11.3)	18 ~ 24	228 (8.9)
25 ~ 44	7917 (24.7)	25 ~ 44	768 (30.0)
45 ~ 64	10045 (31.4)	45 ~ 64	921 (36.0)
65+	10473 (32.7)	65+	643 (25.1)
Sex, N (%)		Sex, N (%)	
Female	17507 (54.6)	Female	1566 (61.2)
Male	14535 (45.4)	Male	944 (38.8)
Race, N (%)		Race, N (%)	
White	20076 (62.7)	White	1834 (71.6)
Nonwhite	4426 (13.8)	Nonwhite	362 (14.1)
Unknown	7540 (23.5)	Unknown	364 (14.2)
Residency, N (%)		Residency, N (%)	
Rural	5518 (17.2)	Rural	491 (19.2)
Urban	23273 (72.6)	Urban	1872 (73.1)
Unknown	3251 (10.2)	Unknown	197 (7.7)
Insurance, N (%)		Insurance, N (%)	
Commercial/Other	8296 (25.9)	Commercial/Other	294 (11.5)
Public	3523 (11.0)	Public	379 (14.8)
Unknown/Uninsured	20223 (63.1)	Unknown/Uninsured	1887 (73.7)
Mortality, N (%)		Mortality, N (%)	
Yes	4689 (14.6)	Yes	566 (22.1)
Age at Death,* Mean (SD)	72.9 (16.5)	Age at Death,* Mean (SD)	64.7 (17.1)
Charlson Comorbidity Index Score 1 Year prior to TBI, Mean (SD)	0.9 (1.6)	Charlson Comorbidity Index Score 1 Year prior to TBI, Mean (SD)	1.9 (2.2)
Charlson Comorbidity Index Score 1 Year prior to TBI, Median (IQR)	0 (0, 1)	Charlson Comorbidity Index Score 1 Year prior to TBI, Median (IQR)	1.0 (0.0, 3.0)
Charlson Comorbidity Condition 1 Year prior to TBI, N (%)		Charlson Comorbidity Condition 1 Year prior to TBI, N (%)	
Yes	12331 (38.5)	Yes	1631 (63.7)
Top 5 Charlson Comorbidity Condition 1 Year prior to TBI, N (%)		Top 5 Charlson Comorbidity Condition 1 Year prior to TBI, N (%)	
Diabetes without chronic complication	4295 (13.4)	Chronic pulmonary disease	707 (27.6)
Chronic pulmonary disease	4036 (12.6)	Diabetes without chronic complication	659 (25.7)
Cerebrovascular disease	3131 (9.8)	Cerebrovascular disease	413 (16.1)
Congestive Heart Failure	2137 (6.7)	Congestive Heart Failure	418 (16.3)
Peripheral vascular disease	1535 (4.8)	Renal disease	344 (13.4)

* Number of patients who died among all cohort participants = 4689

** Number of superutilizers across all visit types who died = 566

**Table 2.** Characteristics of superutilizers based on 75% quartile cutoff point for emergency, inpatient and outpatient services.

	Superutilizer ED		Test statistic (*P* value)	Superutilizer INPATIENT		Test statistic (*P* value)	Superutilizer OUTPATIENT		Test statistic (*P* value)
		
	Yes N = 8331	No N = 23711		Yes N = 9628	No N = 22414		Yes N = 8437	No N = 23605	

**Factors**									
Age at TBI, Mean (SD)	49.5 (20.4)	54.9 (21.6)	20.8 (<0.001)	52.8 (20.8)	53.8 (21.7)	3.8 (<0.001)	56.1 (20.1)	52.6 (21.8)	−13.7 (<0.001)
Length of Stay of the INDEX Inpatient Visit (Days), Mean (SD)	3.6 (11.7)	3.9 (16.7)	1.8 (0.078)	4.5 (12.0)	3.5 (16.8)	−6.0 (<0.001)	3.8 (7.0)	3.8 (17.6)	−0.3 (0.779)
Age at TBI, N (%) 18 ~ 24	1024 (12.3)	2583 (10.9)		1047 (10.9)	2560 (11.4)		563 (6.7)	3044 (12.9)	
25 ~ 44	2607 (31.3)	5310 (22.4)		2521 (26.2)	5396 (24.1)		1901 (22.5)	6016 (25.5)	
45 ~ 64	2707 (32.5)	7338 (31.0)		3051 (31.7)	6994 (31.2)		3041 (36.0)	7004 (29.7)	
65+	1993 (23.9)	8480 (35.8)	477.4 (<0.001)	3009 (31.3)	7464 (33.3)	23.1 (<0.001)	2932 (34.8)	7541 (32.0)	331.2 (<0.001)
Sex, N (%) Female	5005 (60.1)	12502 (52.7)		5648 (58.7)	11859 (52.9)		4852 (57.5)	12655 (53.6)	
Male	3326 (39.9)	11209 (47.3)	134.4 (<0.001)	3980 (41.3)	10555 (47.1)	89.9 (<0.001)	3585 (42.5)	10950 (46.4)	38.1 (<0.001)
Race, N (%) White	6039 (72.3)	14037 (59.2)		6446 (67.0)	13630 (60.8)		4857 (57.6)	15219 (64.5)	
Nonwhite	1499 (18.0)	2927 (12.3)		1602 (16.6)	2824 (12.6)		850 (10.1)	3576 (15.2)	
Unknown	793 (9.5)	6747 (28.5)	1255.6 (<0.001)	1580 (16.4)	5960 (26.6)	416.7 (<0.001)	2730 (32.4)	4810 (20.4)	542.3 (<0.001)
Residency, N (%) Rural	1469 (17.6)	4049 (17.1)		1720 (17.9)	3798 (16.9)		1579 (18.7)	3939 (16.7)	
Urban	6301 (75.6)	16972 (71.6)		7107 (73.8)	16166 (72.1)		6010 (71.2)	17263 (73.1)	
Unknown	561 (6.7)	2690 (11.3)	144.3 (<0.001)	801 (8.3)	2450 (10.9)	51.2 (<0.001)	848 (10.1)	2403 (10.2)	18.0 (<0.001)
Insurance, N (%) Commercial/Other	1062 (12.8)	7234 (30.5)		1704 (17.7)	6592 (29.4)		1903 (22.6)	6393 (27.1)	
Public	1276 (15.3)	2247 (9.5)		1469 (15.3)	2054 (9.2)		727 (8.6)	2796 (11.8)	
Unknown/Uninsured	5993 (71.9)	14230 (60.0)	1081.2 (<0.001)	6455 (67.0)	13768 (61.4)	617.9 (<0.001)	5807 (68.8)	14416 (61.1)	167.4 (<0.001)
Mortality, N (%) Yes	1642 (19.7)	3047 (12.9)	232.2 (<0.001)	1940 (20.2)	2749 (12.3)	335.2 (<0.001)	1390 (16.5)	3299 (14.0)	31.1 (<0.001)
Charlson Comorbidity Index Score during 1 Year prior to TBI, Mean (SD)	1.3 (1.9)	0.8 (1.4)	−22.7 (<0.001)	1.3 (1.9)	0.7 (1.4)	−26.2 (<0.001)	1.6 (2.1)	0.6 (1.2)	−41.4 (<0.001)
Charlson Comorbidity Condition 1 Year prior to TBI, N (%)									
Yes	4143 (49.6)	8197 (34.6)	590.0 (<0.001)	4730 (49.1)	7601 (33.9)	658.6 (<0.001)			
Top 5 Charlson Comorbidity Condition 1 Year prior to TBI, N (%)									
Diabetes without chronic complication	1475 (17.7)	2820 (11.9)	179.4 (<0.001)	1738 (18.1)	2557 (11.4)	256.1 (<0.001)	1977 (23.4)	2318 (9.8)	992.2 (<0.001)
Chronic pulmonary disease	1708 (20.5)	2328 (9.8)	639.1 (<0.001)	1769 (18.4)	2267 (10.1)	417.3 (<0.001)	1782 (21.1)	2254 (9.6)	756.1 (<0.001)
Cerebrovascular disease	983 (11.8)	2148 (9.1)	52.5 (<0.001)	1229 (12.8)	1902 (8.5)	139.9 (<0.001)	1285 (15.2)	1846 (7.8)	387.1 (<0.001)
Congestive Heart Failure	855 (10.3)	1282 (5.4)	233.6 (<0.001)	1052 (10.9)	1085 (4.8)	400.7 (<0.001)	1034 (12.3)	1103 (4.7)	574.1 (<0.001)
Peripheral vascular disease	493 (5.9)	1042 (4.4)	31.4 (<0.001)	638 (6.6)	897 (4.0)	101.7 (<0.001)	725 (8.6)	810 (3.4)	363.1 (<0.001)

**Table 3.** Odds ratios from multivariate logistic regression for being a superutilizer based on 75% quartile cutoff point.

Utilization	Variable	Level of Variable	Reference of Variable	Odds Ratio 95% CI	P-value

**Emergency**	Healthcare utilization (no. Visits) 1 year prior to index TBI event			1.44 (1.42, 1.46)	<.001
	Age	18 ~ 24	25 ~ 44	0.88 (0.79, 0.97)	0.010
		18 ~ 24	45 ~ 64	1.07 (0.97, 1.19)	0.189
		18 ~ 24	65+	1.39 (1.25, 1.54)	<.001
		25 ~ 44	45 ~ 64	1.23 (1.13, 1.33)	<.001
		25 ~ 44	65+	1.58 (1.46, 1.72)	<.001
		45 ~ 64	65+	1.29 (1.20, 1.39)	<.001
	Charlson Comorbidity during 1 Year Prior	Unit = 1		1.35 (1.27, 1.45)	<.001
	Sex	Female	Male	1.07 (1.01, 1.13)	0.030
	Residency	Rural	Urban	0.97 (0.89, 1.05)	0.402
	Race	Nonwhite	White	1.07 (0.99, 1.16)	0.085
	Insurance	Commercial/Other	Public	0.55 (0.49, 0.61)	<.001
**Inpatient**	Healthcare utilization (no. Visits) 1 year prior to index TBI event			1.31 (1.29, 1.33)	<.001
	Age	18 ~ 24	25 ~ 44	0.83 (0.76, 0.91)	<.001
		18 ~ 24	45 ~ 64	0.83 (0.76, 0.92)	<.001
		18 ~ 24	65+	0.90 (0.81, 0.99)	0.026
		25 ~ 44	45 ~ 64	1.01 (0.94, 1.08)	0.888
		25 ~ 44	65+	1.08 (1.00, 1.16)	0.041
		45 ~ 64	65+	1.07 (1.01, 1.14)	0.032
	Charlson Comorbidity during 1 Year Prior	Unit = 1		1.44 (1.36, 1.53)	<.001
	Sex	Female	Male	0.99 (0.94, 1.05)	0.809
	Residency	Rural	Urban	1.07 (1.00, 1.14)	0.062
	Race	Nonwhite	White	1.13 (1.04, 1.21)	0.002
	Insurance	Commercial/Other	Public	0.57 (0.52, 0.63)	<.001
**Outpatient**	Healthcare utilization (no. Visits) 1 year prior to index TBI event			1.07 (1.07, 1.07)	<.001
	Age	18 ~ 24	25 ~ 44	0.67 (0.60, 0.75)	<.001
		18 ~ 24	45 ~ 64	0.67 (0.60, 0.75)	<.001
		18 ~ 24	65+	0.83 (0.74, 0.94)	0.002
		25 ~ 44	45 ~ 64	1.00 (0.92, 1.08)	0.914
		25 ~ 44	65+	1.24 (1.14, 1.35)	<.001
		45 ~ 64	65+	1.25 (1.16, 1.34)	<.001
	Charlson Comorbidity during 1 Year Prior	Unit = 1		1.38 (1.29, 1.47)	<.001
	Sex	Female	Male	0.90 (0.84, 0.95)	<.001
	Residency	Rural	Urban	1.04 (0.97, 1.12)	0.268
	Race	Nonwhite	White	0.79 (0.72, 0.87)	<.001
	Insurance	Commercial/Other	Public	1.07 (0.96, 1.20)	0.234
